# Psychological burden of hearing-impaired children and their parents through the COVID-19 pandemic

**DOI:** 10.3389/fpubh.2024.1403729

**Published:** 2024-09-17

**Authors:** Donglei Li, Ken Lin, Xinru Cen, Yuwei Fan, Liping Hong, Zhao Wu, Wenliang Chen, Xinqi Zhong

**Affiliations:** ^1^Department of Pediatrics, Guangdong Provincial Key Laboratory of Major Obstetric Diseases; Guangdong Provincial Clinical Research Center for Obstetrics and Gynecology; Guangdong-Hong Kong-Macao Greater Bay Area Higher Education Joint Laboratory of Maternal-Fetal Medicine; The Third Affiliated Hospital of Guangzhou Medical University, Guangzhou, China; ^2^Clinical Medical College, Guangzhou Medical University, Guangzhou, China; ^3^Scientific Research Center, The Second Affiliated Hospital of Guangdong Medical University, Zhanjiang, China

**Keywords:** hearing loss, mental health, COVID-19, anxiety, depression

## Abstract

**Objective:**

Childhood hearing impairment has potential repercussions on the mental well-being of both children and their parents. As a vulnerable population in accessing health care services, they may face specific challenges, especially during the COVID-19 pandemic. This cross-sectional study aims to investigate the association between childhood hearing impairment and the mental health of children and their parents, and to assess health care utilization of hearing-impaired children and its impact on mental outcomes for both during the COVID-19 pandemic.

**Methods:**

Using the National Health Interview Survey (NHIS) database, we analyzed data for 15,989 children aged 5–17 and their corresponding parents. The correlations between childhood hearing impairment and mental outcomes were examined using logistic regression models. The 2020 (quarter 3 and quarter 4)-2021 NHIS data was singled out and re-analyzed, focusing on the utilization of medical care during the COVID-19 pandemic.

**Results:**

After accounting for covariates, hearing-impaired children exhibited a higher frequency of anxiety (OR 2.33, 95% CI 1.79–3.02) or depression (OR 2.14, 95% CI 1.59–2.88). Parents of hearing-impaired children had significantly higher odds of a higher frequency of anxiety (OR 1.55, 95% CI 1.20–2.01) or depression (OR 1.73, 95% CI 1.30–2.29). Interaction effect of hearing impairment with survey year on parents’ mental health outcomes was observed (*p* for interaction <0.1). Children with hearing loss had higher odds of reporting delayed medical care (OR 2.00, 95% CI 1.11–3.59) or canceled medical care (OR 1.96, 95% CI 0.98–3.96, *p* = 0.059) due to the pandemic. Delayed medical care (OR 12.41, 95% CI 2.78–55.46) or canceled medical care (OR 6.26, 95% CI 1.28–30.75) due to the COVID-19 pandemic significantly contributed to the increase of anxiety frequency in hearing-impaired children.

**Conclusion:**

Childhood hearing impairment exhibits a substantial impact on children’s and parental mental health, which is further exacerbated by the COVID-19 pandemic. Families of hearing-impaired children appear to be in a vulnerable position during public health emergencies such as the COVID-19 pandemic, which can further exacerbate their mental outcomes.

## Introduction

Hearing impairment stands as one of the most common developmental disorders in children, profoundly affecting not only various facets of a child’s life (1), but also their mental well-being (2). Hearing loss increases the likelihood of reporting child behavioral diagnoses, behavior issues, and socioemotional difficulties (3). Raising a hearing-impaired child also presents considerable challenges and complexities. For instance, parents need to update to the latest knowledge about hearing loss, and health care services (mainly aural habilitation) and deal with communication difficulties (4). Additionally, the financial burden related to sensory devices and follow-up care (5), and the stress related to the behavioral problems of children (6) should be considered. Some research has indicated that the stress experienced by families raising children with hearing impairment can be bidirectional, affecting both the children’s mental health and their parents (7–10) and may lead to a vicious cycle (11). While others suggest that children’s or parental mental well-being is not significantly associated with hearing impairment in children (9, 11, 12). It is important to note that variables related to the parents and family, such as race, gender, and socioeconomic condition (7, 13–16), are correlated with the manifestation of behavioral and emotional disorders in hearing-impaired children and their parents, which may contribute to conflicting results. Given that many of these conclusions have arisen from small-sample or single-center analyses that cannot adequately represent a broader population, there is a compelling need for more comprehensive, larger-scale studies and in-depth analyses to clarify the specific effects of hearing impairment on children’s and their parents’ mental health.

The Corona virus disease 2019 (COVID-19) pandemic was a global health event that had far reaching consequences, causing a detrimental psychological impact on diverse populations across the world (17, 18). The psychological burden of COVID-19 varied among individuals, and certain groups were identified as being at a higher risk for developing psychological problems due to the pandemic, warranting additional attention, these include hearing-impaired children and their parents (19–22). Studies have revealed a significant decline in the mental well-being of hearing-impaired children and their caregivers during the peak stage of the COVID-19 pandemic. Specifically, a study conducted in Iran showed that more than one third of hearing-impaired adolescents experienced anxiety disorder during the COVID-19 pandemic (23). Another study of parents of hearing-impaired children showed that 55% of parents showed clinically high anxiety symptoms during the pandemic, and 16% recognized an increase in depressive symptoms, which are three to four times the prevalence in the general population, respectively (24). During the pandemic, reports also emerged concerning heightened parental stress, deteriorating mental health, and an increased incidence of child maltreatment (25).

It is essential to thoroughly investigate the association between childhood hearing impairment and adverse mental outcomes of children and their parents, especially during the COVID-19 pandemic. Additionally, though the inequality of hearing-impaired population in health care services during the COVID-19 pandemic was reported (26), there has been a lack of specific research focusing on hearing-impaired children. Whether the increased need for health services due to speech rehabilitation and other needs for hearing-impaired children may act as a significant stressor of mental health is also a question that needs to be explored. This can help better understand their situation and provide more targeted and comprehensive social support for them during public health emergencies such as the COVID-19 pandemic. From this aspect, this study aimed to investigate the impact of childhood hearing impairment on children’s and their parents’ mental health based on a large cohort from the National Health Interview Survey (NHIS). We also explored the challenges experienced by families with hearing-impaired children while acquiring the health care service during the COVID-19 pandemic to shed further light on the psychological burden faced by our target population over the past few years.

## Methods

### Data source and selection

**Figure 1:**
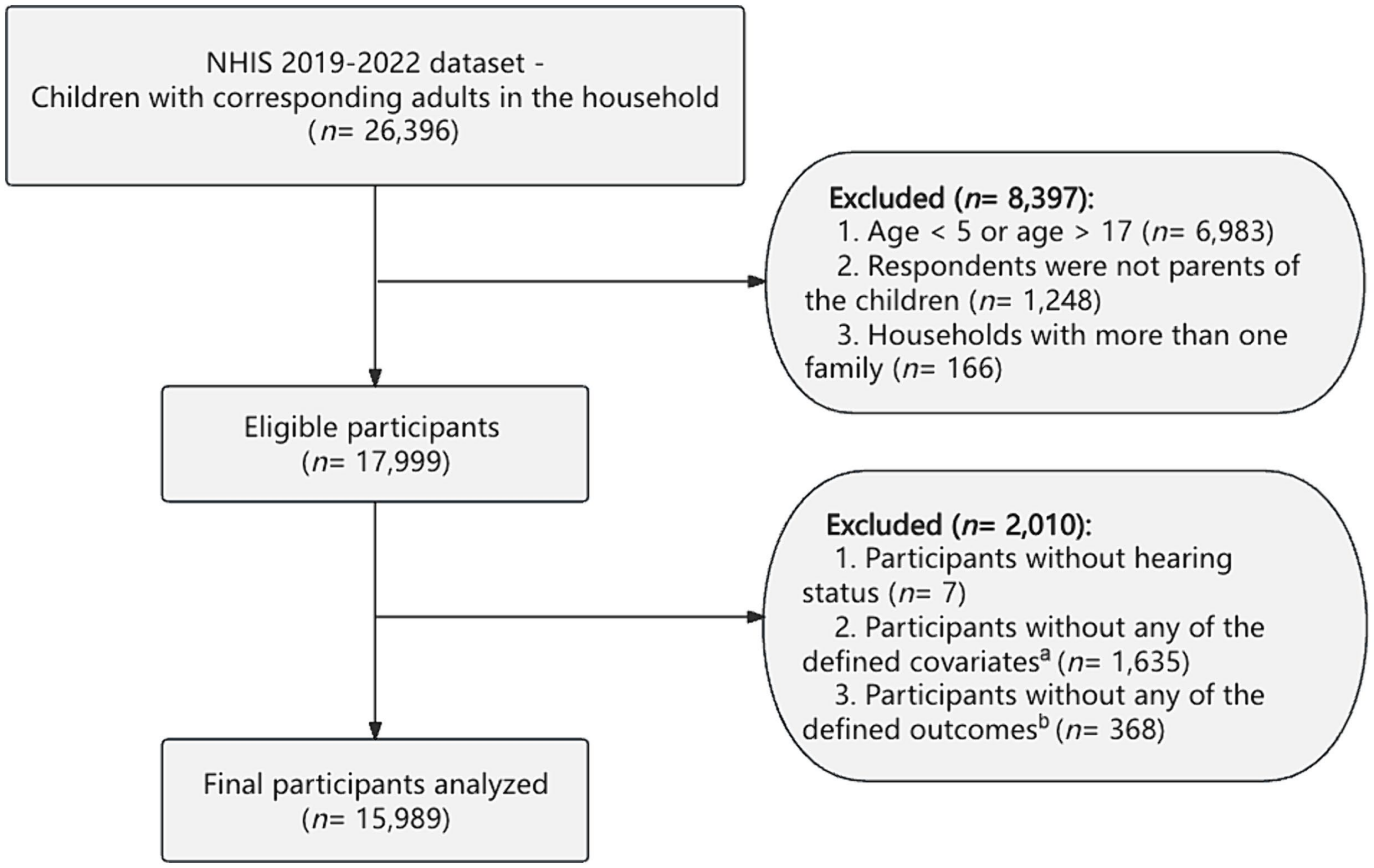
The overall design of this study. NHIS, National Health Interview Survey. ^a^Defined covariates included age of children, sex of children, race group of children, the number of adults in the family, the number of children in the family, and ratio of family income to poverty threshold (PIR), the age of the parents, sex of the parents, educational level of the parents, race group of the parents, and the presence of hearing impairment in the parents. ^b^Defined outcomes included children’s outcomes (higher frequency of anxiety, higher frequency of depression) and parental outcomes (anxiety disorder, depression disorder, higher frequency of anxiety, higher frequency of depression).

The data were derived from the National Health Interview Survey (NHIS),^1^ a broad health survey aimed at the United States civilian, non-institutionalized population, ranging from 2019 to 2022. The NHIS employs a systematic approach to sample households, wherein each sampled household provides detailed health information for one adult and one child. In the case of children, a knowledgeable adult in the household, typically a parent, responds to questions about the child’s health. In this study, we included households with children between the ages of 5 and 17. This age was chosen because these children are typically enrolled in school, making them particularly susceptible to the impacts of the COVID-19 outbreak and the relevant lockdown policies. We excluded households where the respondents were not biological, adoptive, or stepparents of the children. Households with more than one family were also excluded from the analysis. We also omitted households with missing data concerning the hearing status, age, sex, race, number of adults in the household, and number of children in the household. In cases where the mother or father was present in the household but data on the parent’s age, sex, education, income-to-poverty threshold ratio, or hearing status was missing, we excluded those observations. A flow chart of our study design was presented in Figure 1. A child or a parent was categorized as having hearing impairment if they reported experiencing “a little trouble hearing,” “moderate trouble,” “a lot of trouble,” or “deaf” when asked about the ability to hear without the use of hearing aids or other listening devices.

### Definition of mental outcomes

Mental outcomes of children were assessed and reported by their parents according to their answers to the following two questions: “How often does this child seem anxious, nervous, or worried?” and “How often does this child seem very sad or depressed?” The answer options were: “Daily,” “Weekly,” “Monthly,” “A few times a year,” or “Never.” Responses of “Daily,” “Weekly,” or “Monthly,” were considered as a higher frequency of anxiety or depression while responses of “A few times a year” or “Never” were defined as a lower frequency of anxiety or depression.

For parents’ mental outcomes, they were presented with two key questions: “Have you ever been told by a doctor or other health professional that you had any type of anxiety disorder?” and “Have you ever been told by a doctor or other health professional that you had any type of depression?” Whether they had anxiety or depression disorder was based on whether the parent answered “Yes,” or “No,” to the question. To further assess the more recent status of mental health, the assessment of anxiety and depression frequency was also included. Participants’ responses to the questions “How often do you feel worried, nervous or anxious?” and “How often do you feel depressed?” The answer options were: “Daily,” “Weekly,” “Monthly,” “A few times a year,” or “Never.” Responses of “Daily,” “Weekly,” or “Monthly,” were classified as a higher frequency of anxiety or depression, while responses of “A few times a year,” or “Never,” were defined as a lower frequency of anxiety or depression. More details about the raw questionnaire were shown in Supplementary methods.

### Determination of covariates

In this study, several covariates were considered to account for the potential confounding factors and to better illustrate the relationships under investigation. The covariates included children’s characteristics, family composition, parents’ characteristics, and survey year. The children’s characteristics included the children’s sex, race (White only, Black/African American only, others), and age (≤12 years, >12 years). Regarding the potential influence of family economic factors and other members of the family, we controlled for number of adults in the family (1, 2, 3+), the number of children in the family (1, 2, 3+), and ratio of family income to poverty threshold, (<2, 2–3.99, ≥4). For parents’ characteristics, we considered their sex, race (White only, Black/African American only, others), age (≤40 years, >40 years), educational level (High school and below, Associate degree or some college, Bachelor’s degree and above), and hearing status (No impairment, Hearing impairment). Considering the potential impact of the COVID-19 pandemic on clinical outcomes related to mental issues, we included the survey year as a covariate, which was grouped into “before COVID-19 (2019),” “peak stage of COVID-19 (2020–2021),” and “late stage of COVID-19 (2022).”

### Assessment of health care service during the pandemic

The 2020 (quarter 3 and quarter 4)-2021 NHIS data included variates concerning health care utilization during the COVID-19 pandemic. Children’s delayed medical care due to COVID-19 was examined by asking their parents “Was there any time when this child delayed getting medical care because of the coronavirus pandemic?” Additionally, children’s absence of medical care was assessed through the question “Was there any time when this child needed medical care for something other than coronavirus, but did not get it because of the coronavirus pandemic?” The answer options to these questions were: “Yes,” or “No.”

### Statistical analysis

Analyses were conducted using R software (4.2.1). To reduce the possibility of selection bias, these analyses took into account the complex sampling design of the NHIS, which allows for the incorporation of the strata, cluster, and final weight into the data analysis. This approach ensures the results are representative of the broader population. All data were weighted using the weight of the child (WTFA_C). Pearson χ^2^ statistics was used to compare the basic characteristics between children with and without hearing impairment. To eliminate the confounding bias, we conducted both univariate and multivariate logistic regression analyses to evaluate the relationships between childhood hearing impairment and the outcomes related to children’s and parental anxiety and depression. The odds ratio indicates how many times certain risk is for children with hearing impairments compared to children without hearing impairments, or for parents of children with hearing impairments compared to parents of children without hearing impairments. The multivariate models were adjusted for covariates mentioned above, allowing for the isolation of the specific effects of hearing impairment while controlling for potential confounding variables. To further reduce the confounding bias and explore the heterogeneity of the study population, subgroup analyses were conducted using multivariate logistic regression, incorporating the covariates mentioned above to evaluate the potential differential effects of hearing impairment on the outcomes within different demographic and contextual subgroups. Forest plots were used to illustrate these interactive analyses. Furthermore, the 2020 (quarter 3 and quarter 4)-2021 NHIS data with responses concerning the COVID-19 pandemic was singled out and re-analysis accounted for the complex weighting variable of the surveys. Logistic regressions controlling for covariates were also used to test the association between childhood hearing loss and health care utilization, as well as the association between health care utilization and mental outcomes in hearing-impaired children. A *p* value <0.05 in baseline analysis was considered statistical significance, while a *p* for interaction <0.1 in subgroup analysis was also considered to have an interaction effect.

## Results

### Study population of 2019–2022 NHIS

Our unweighted sample included 15,989 children who were evaluated by means of questionnaires completed by their parents, 349 were reported hearing impairment and 15,640 were not reported hearing impairment. In the weighted sample that represented a population of about 37.7 million, more than 2% of the children were identified as suffering from hearing impairment. Among the hearing-impaired children, 12.5% were Black, 74% were White, and 13.5% were individuals of other races. For parents of hearing-impaired children, females accounted for 64.7% while males for 35.3% (Table 1). Households with hearing-impaired children were more likely to have an income-to-poverty ratio < 2 compared to those with normal-hearing children (43.5% vs. 34.2%, *p* < 0.05, Table 1). Furthermore, parents of children with hearing impairment were more likely to be under the age of 40 (59.9% vs. 50.4%, *p* < 0.01), be with an educational level of high school or below (40.2% vs. 29.7%, *p* < 0.01), and report hearing difficulties (20.7% vs. 8.3%, *p* < 0.001) than parents of children without hearing impairment (Table 1).

**Table 1:** Characteristics of the study population, 2019–2022 NHIS.

Variables	Without hearing impairment	With hearing impairment	*p* value
Total (%, weighted *n*)	97.8 (36874962.6)^a^	2.2 (828364.8)^a^	
Year of interview (%)			0.787
Before COVID-19	25.3	26.7	
Peak stage of COVID-19	50.0	50.1	
Late stage of COVID-19	24.8	23.1	
**Parental characteristics**			
Age (%)			0.001**
≤ 40	50.4	59.9	
> 40	49.6	40.1	
Sex (%)			0.066
Male	40.9	35.3	
Female	59.1	64.7	
Educational level (%)			0.001**
High school or below	29.7	40.2	
Associate degree or some college	29.0	25.6	
Bachelor’s degree or above	41.3	34.2	
Race group (%)			0.671
White only	75.7	77.8	
Black/African American only	14.6	12.7	
Others^b^	9.6	9.5	
Hearing impairment (%)			<0.001***
No impairment	91.3	79.7	
Hearing impairment	8.7	20.3	
**Family’s characteristics**			
PIR (%)			0.006**
< 2	34.2	43.5	
2–4	30.1	25.2	
> 4	35.7	31.3	
Number of adults in the family (%)			0.555
1	17.7	18.1	
2	64.9	62.0	
3+	17.4	19.8	
Number of children in the family (%)			0.537
1	20.7	21.9	
2	39.9	36.6	
3+	39.3	41.5	
**Children’s characteristics**			
Age (%)			0.509
≤ 12	61.3	63.4	
> 12	38.7	36.6	
Sex (%)			0.230
Male	51.3	54.9	
Female	48.7	45.1	
Race group (%)			0.697
White only	72.9	74.0	
Black/African American only	14.3	12.5	
Others^b^	12.7	13.5	

PIR, Ratio of family income to poverty threshold. *p < 0.05. **p < 0.01. ***p < 0.001.

a A total of 17,999 families were eligible for inclusion, of which 2010 (11.17%) were excluded due to incomplete data. Unweighted number of families with hearing impaired children was 349 and the number of families without hearing impaired children was 15,640.

b Other race groups include responses of “Asian only,” “American Indian and Alaska Native [AIAN] only,” “AIAN and any other group,” and “Other single and multiple races”.

#### Mental outcomes

The results of both univariate and multivariable logistic regression analyses were shown in Table 2, exploring the relationship between hearing impairment in children and various mental outcomes. In Model 3, which adjusted for all defined covariates, childhood hearing impairment exhibited independent associations with all defined outcomes. Hearing-impaired children tend to have higher frequency of anxiety disorder (OR 2.33, 95% CI 1.79–3.02) and depression disorder (OR 2.14, 95% CI 1.59–2.88) compared to normal hearing children. Parents whose children suffered from hearing impairment had significantly higher odds of experiencing anxiety disorder (OR 1.90, 95% CI 1.44–2.49) and depression disorder (OR 1.72, 95% CI 1.30–2.29) in comparison to those without hearing impairment children. Furthermore, they showed a higher frequency of anxiety (OR 1.55, 95% CI 1.20–2.01) and depression (OR 1.73, 95% CI 1.30–2.29).

**Table 2:** Univariate and multivariate logistic regression results for children’s and parental outcomes.

	Hearing impairment versus No hearing impairment		
Outcomes	Crude Model^a^	Model 1^b^	Model 2^c^
	OR (95%CI)	OR (95%CI)	OR (95%CI)
Children’s outcomes			
Higher frequency of anxiety	2.22 (1.72–2.86)	2.41 (1.86–3.11)	2.33 (1.79–3.02)
Higher frequency of depression	2.16 (1.60–2.91)	2.27 (1.68–3.07)	2.14 (1.59–2.88)
Parental outcomes			
Anxiety disorder	2.21 (1.71–2.86)	2.19 (1.69–2.83)	1.90 (1.44–2.49)
Higher frequency of anxiety	1.67 (1.29–2.17)	1.70 (1.31–2.20)	1.55 (1.20–2.01)
Depression disorder	2.06 (1.57–2.69)	2.02 (1.53–2.66)	1.72 (1.30–2.29)
Higher frequency of depression	2.07 (1.57–2.73)	2.01 (1.52–2.66)	1.73 (1.30–2.29)

OR, Odds ratio; CI, Confidence interval.

a Unadjusted for any covariate.

b Adjusted for year of interview, age of children, sex of children, race group of children, the number of adults in the family, the number of children in the family, and ratio of family income to poverty threshold (PIR).

c Building upon Model 1 with additionally incorporating the age of the parents, sex of the parents, educational level of the parents, race group of the parents, and the presence of hearing impairment in the parents.

#### Subgroup analysis

**Figure 2:**
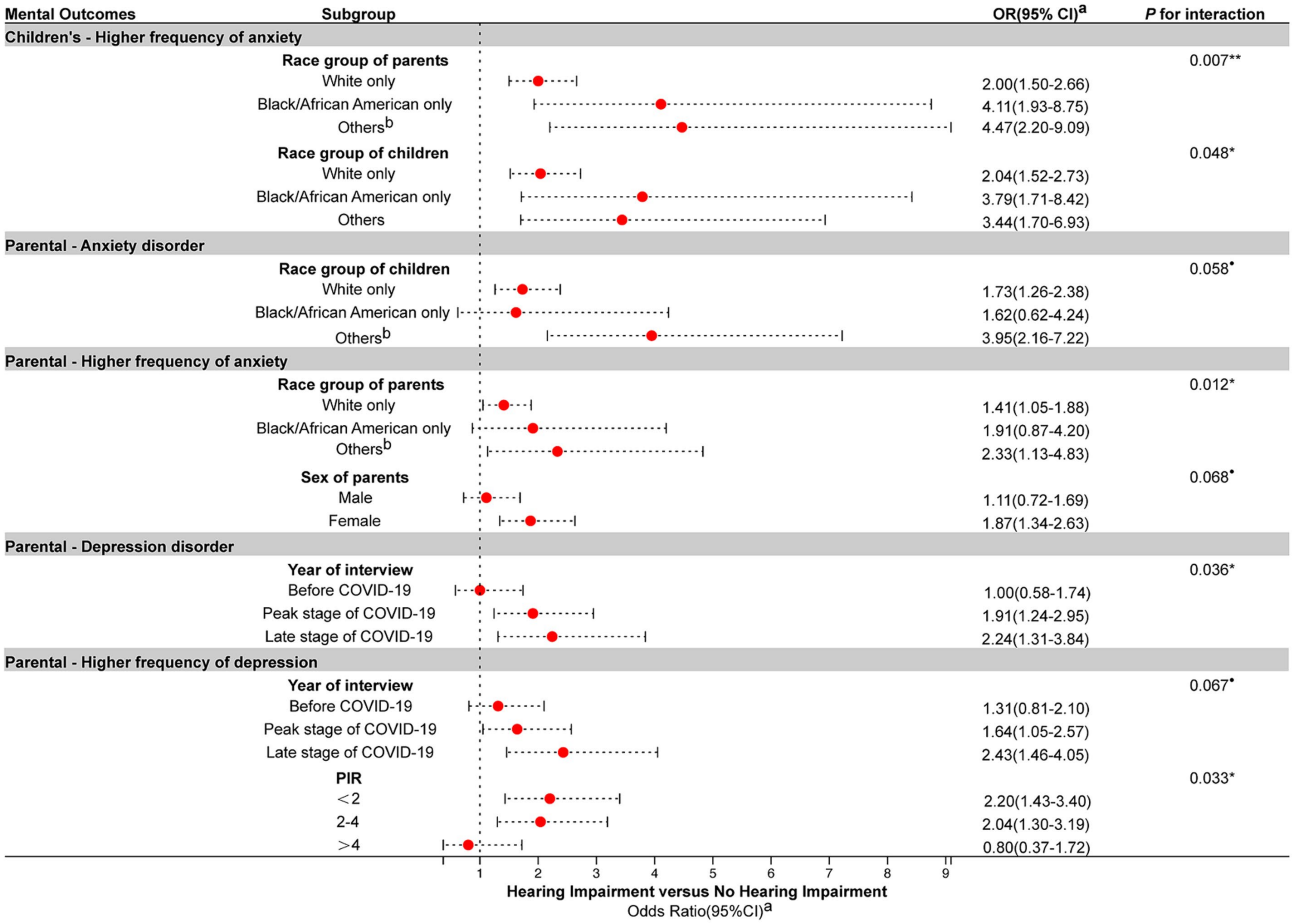
Association between childhood hearing impairment and children’s and parental outcomes in subgroup analyses. OR, Odds ratio; CI, Confidence interval; PIR, Ratio of family income to poverty threshold. ^a^Adjusted for age of children, sex of children, race group of children, the number of adults in the family, the number of children in the family, and ratio of family income to poverty threshold (PIR), the age of the parents, sex of the parents, educational level of the parents, race group of the parents, and the presence of hearing impairment in the parents. ^b^Other race groups include responses of “Asian only,” “American Indian and Alaska Native [AIAN] only,” “AIAN and any other group,” and “Other single and multiple races.” Only socioeconomic demographic factors that exhibit significant interaction effect with childhood hearing loss on children’s and parental anxiety and depression outcomes (all *p* for interaction <0.1) were selected and depicted. Comprehensive interaction results encompassing all subgroups for each parental outcome are presented in Supplementary Tables 1–6. ^•^*p* < 0.1. *p < 0.05. **p < 0.01.

We identified several socioeconomic demographic factors exhibiting significant interaction effects with childhood hearing loss on children’s and parental anxiety and depression outcomes (all *p* for interaction <0.1), which were selected and depicted in Figure 2. Additionally, comprehensive interaction results encompassing all subgroups for each mental outcome are presented in Supplementary Tables 1–6. Both children’s and parents’ race groups exhibited a significant interaction effect with childhood hearing loss on children’s anxiety frequency (all *p* for interaction <0.05). Anxiety frequency of black/African American children (OR 3.79, 95% CI 1.71–8.42) and children from other race groups (OR 3.44, 95% CI 1.70–6.93) was more likely to be increased by their hearing impairment compared to White children. For parental anxiety disorder, a distinct interaction effect between childhood hearing loss and the race group of children was observed (*p* for interaction = 0.058). Furthermore, for a higher frequency of parental anxiety, interaction effects with childhood hearing loss were noted, involving both the race group of parents (*p* for interaction = 0.012) and the sex of parents (*p* for interaction = 0.068). Additionally, the ratio of family income to poverty threshold (PIR) was also found to be a significant interaction term with childhood hearing impairment on a higher frequency of parental depression (*p* for interaction = 0.033).

The association between childhood hearing loss and parental depression disorder was found to be contingent on the year of interview, with distinctive odds ratios observed across different time periods: before COVID-19 (OR 1.00, 95% CI 0.58–1.74), the peak stage of COVID-19 (OR 1.91, 95% CI 1.24–2.95), and late stage of COVID-19 (OR 2.24, 95% CI 1.31–3.84). Similarly, the interaction effect between childhood hearing impairment and the year of interview on a higher frequency of parental depression was observed (*p* for interaction = 0.067).

### Study population of 2020 (quarter 3 and quarter 4)-2021 NHIS

To further investigate the impact of the COVID-19 pandemic on hearing-impaired children and their families, the 2020 (quarter 3 and quarter 4)-2021 NHIS data was singled out and re-analyzed, focusing on the utilization of medical care during the pandemic and its impact on the mental well-being of families with hearing-impaired children. Baseline characteristics of this population were shown in Supplementary Table 7. In this study cohort, 2.1% of the participants were hearing-impaired children, whose parents experienced a higher prevalence of hearing difficulties (*p* < 0.001). They also showed a difference in the number of children in the family between normal-hearing children (*p* = 0.047).

### Health care utilization during the COVID-19 pandemic

After controlling for covariates, children with hearing loss were more likely to have delayed their medical care because of the COVID-19 pandemic (OR 2.00, 95% CI 1.11–3.59, Table 3). Hearing impaired children also showed a trend of being more likely to report an inability to access medical care in comparison to those without hearing impairment, though at the verge of significance (OR 1.96, 95% CI 0.98–3.96, *p* = 0.059, Table 3).

**Table 3:** Univariate and multivariate logistic regression models of delayed and absent medical care because of the COVID-19 pandemic among children during the peak stage of COVID-19 (2020 [quarter 3 and quarter 4]-2021).

	Delayed medical care		Absent medical care	
	Crude model^a^	Model 1^b^	Crude model^a^	Model 1^b^
	OR (95%CI)	OR (95%CI)	OR (95%CI)	OR (95%CI)
Hearing status				
Without hearing impairment	Reference	Reference	Reference	Reference
With hearing impairment	1.93 (1.07–3.47)	2.00 (1.11–3.59)	1.97 (0.97–3.97)	1.96 (0.98–3.96)

OR, Odds ratio; CI, Confidence interval.

a Unadjusted for any covariate.

b Adjusted for age of children, sex of children, race group of children, the number of adults in the family, the number of children in the family, and ratio of family income to poverty threshold (PIR).

After adjusting for covariates, delayed medical care due to the COVID-19 pandemic strongly contributed to the increase of anxiety frequency in hearing-impaired children (OR 12.41, 95% CI 2.78–55.46, Table 4). Additionally, hearing-impaired children who had experienced an inability to access medical care due to the COVID-19 pandemic showed a higher frequency of anxiety (OR 6.26, 95% CI 1.28–30.75, Table 4). No significant association was found between other defined outcomes and delayed/canceled medical care due to the COVID-19 pandemic in hearing-impaired children.

**Table 4:** Univariate and multivariate logistic regression results for children’s and parental outcomes of delayed and absent medical care because of the COVID-19 pandemic in hearing impaired children.

	Delayed medical care versus No delayed medical care		Absent medical care versus No Absent medical care	
	Crude model^a^	Model 1^b^	Crude model^a^	Model 1^b^
	OR (95%CI)	OR (95%CI)	OR (95%CI)	OR (95%CI)
Children’s outcomes				
Higher frequency of anxiety	9.58 (2.43–37.74)	12.41 (2.78–55.46)	5.66 (1.16–27.53)	6.26 (1.28–30.75)
Higher frequency of depression	2.49 (0.75–8.29)	2.79 (0.63–12.32)	1.83 (0.43–7.81)	2.16 (0.36–12.88)
Parental outcomes				
Anxiety disorder	1.04 (0.34–3.11)	1.07 (0.32–3.55)	1.40 (0.32–6.05)	1.13 (0.24–5.38)
Higher frequency of anxiety	1.31 (0.43–3.99)	1.12 (0.32–3.95)	1.05 (0.27–4.15)	0.66 (0.15–3.01)
Depression disorder	1.94 (0.66–5.71)	2.21 (0.62–7.93)	3.01 (0.76–11.95)	3.71 (0.68–20.2)
Higher frequency of depression	1.64 (0.56–4.82)	2.08 (0.58–7.44)	2.84 (0.72–11.13)	3.39 (0.58–19.61)

OR, Odds ratio; CI, Confidence interval.

a Unadjusted for any covariate.

b Adjusted for age of children, sex of children, race group of children, the number of adults in the family, the number of children in the family, and ratio of family income to poverty threshold (PIR).

## Discussion

In this study, we analyzed data from 2019 to 2022, consisting of 15,989 samples of family, followed by a re-analysis of the 2020 (quarter 3 and quarter 4)-2021 NHIS data. Our study reveals a strong association between childhood hearing impairment and the reported rates and frequency of both children’s and parental anxiety and depression. This association remained significant even after adjusting for the demographic and socioeconomic characteristics of both parents and children.

The notion that hearing impairment may have an adverse impact on children’s mental well-being has been validated by numerous studies, reporting a strong link between hearing impairment and anxiety, depression, and other mental problems in children (8, 27, 28). Using a nationally representative survey, we again demonstrated this observation. Some researchers attributed the higher rates of depression in hearing-impaired children to communication barriers in the auditory world and adverse experiences associated with stigma and discrimination (29). Another study supported this idea from the perspective of emotional and behavioral difficulties in hearing-impaired children (30). Additionally, the psychological burden of parents with hearing-impaired children was noteworthy, indicating parental concerns about the children’s future due to decreased communication skills and delayed language development (31). The limited social support for parents of hearing-impaired children was also a concern (12). A longitudinal study has revealed that nearly half of children with hearing impairments experience hearing deterioration (32), which, in turn, can lead to feelings of guilt and self-blame among parents as they deal with the progression of their children’s disability.

It is notable that parents’ psychological well-being can profoundly affect their children’s overall welfare. Studies have shown that elevated levels of parental stress are associated with children’s language delays, behavioral problems and the functional hearing ability of children, especially in noisy environments (10, 33, 34). Additionally, when parents reported experiencing mild to severe depressive symptoms, there was a significant decrease in their children’s usage time for hearing aids (35), implying worse treatment compliance and prognosis. Therefore, it is a potential area for future researchers and social workers to construct a comprehensive caring system involving psycho-social support for families with hearing-impaired children, stressing more on the mental well-being of all family members.

To further identify the high-risk pool of families whose mental well-being is more susceptible to childhood hearing loss, we conducted subgroup analysis. Several factors, including race group, the sex of parents, and PIR, were found to be involved in the relationship. Specifically, hearing-impaired families of races other than white and black may experience unique challenges, like discrimination and communication difficulties caused by cultural differences or other factors. Regarding the parental sex, a recent study showed that mothers and fathers did show differences in the practical subdomains of parental stress, implying that hearing impairment in children has a varying psychological impact on fathers and mothers, and mothers often reported poorer mental health than fathers (36), a trend consistent with the results in our study. The influence of PIR on parental depression is well-established. Income is a recognized correlate of depression (37), and low family income has been found to be associated with lifelong mental disorders and suicide attempts (15), especially during the pandemic period (16). These findings helped us further locate those families that are more vulnerable to children’s hearing impairment, enabling us to implement more targeted health care strategies to enhance the welfare of the disabled.

Interestingly, we found that the COVID-19 pandemic was also involved in the interaction. To be specific, we observed that the risk of depression, along with high-frequency depression, among parents of children with hearing impairment experienced significant growth at the peak stage of the COVID-19 pandemic and did not recover over time; instead, it continued to increase in the post-COVID-19 era. Given previous articles have suggested a correlation between children’s developmental outcomes and parents’ poor mental health outcomes (10, 12, 38), we speculate that COVID-19 may have a long-term negative effect on parents’ mental well-being by affecting the development and recovery of hearing-impaired children. If the pandemic and associated lockdown policies interfere with hearing impaired children’s access to treatment and communication, leading to poorer child development outcomes, parents are inevitably confronted with more serious parenting challenges. This circumstance may offer one explanation for the sustained increase in their reports of depression.

Consequently, we focused on the utilization of medical care among hearing-impaired children during the COVID-19 pandemic, as well as its association with defined mental outcomes of their families. Results showed that children with hearing impairment were more likely to suffer medical delays and cancelations due to the COVID-19 pandemic than normal-hearing children. Additionally, we observed that hearing-impaired children with medical delays or cancelations due to the COVID-19 pandemic experienced an increase in the incidence and frequency of anxiety. It was in line with our assumption, regarding the fact that families with hearing-impaired children are often vulnerable sections of society. Previous research had also reported that adults with hearing loss were more likely to fail to access health utilization during the COVID-19 pandemic, and had called for supportive policies for hearing-impaired populations (26). For hearing impaired children, considering their special needs for routine treatments such as speech rehabilitation, it could lead to poorer mental outcomes if the pandemic prevented them from accessing relevant medical services.

Therefore, future studies should focus more on the long-term effects of COVID-19 on children’s and parents’ psychological health, such as anxiety and depression disorders. Though lockdown policies and economic contraction caused by the COVID-19 pandemic may eventually fade, it is crucial to determine to what extent the potential absence of health care during the pandemic has affected the long-term psychological well-being of families with hearing-impaired children. This may help formulate more targeted policies to mitigate such impacts in similar public health emergencies in the future.

In addition, since we have found that children’s hearing impairment was associated with an increased risk of mental disorders for both children and parents in this study, it is a worthy topic for future research whether their anxiety or depression will mutually influence and further lead to worse health outcomes, especially in high-pressure atmosphere such as public health emergencies. For families with children with hearing impairment, a more vulnerable group, social workers, primary health care units, and specialists should work closely and pay attention to the whole process of their mental health from screening, and intervention to follow-up of the families. And establish a more accessible pathway of health care service for this population in public health emergencies and carry out continuous follow-up through telemedicine and other means to promote the equality of health care services of this population.

### Strengths and limitations

This study utilized a large nationally representative database to help control for various confounding factors and explore the relationship between children’s hearing impairment and caregivers’ mental health. Furthermore, the influence of the COVID-19 pandemic on health care utilization of hearing-impaired children, and its adverse impact on caregivers were considered. However, several limitations should be considered in our study. First, it is important to realize that this is a cross-sectional study, relying on the analysis of family samples randomly selected between 2019 and 2022. Therefore, the NHIS data can only be used as a reference for understanding the associations between childhood hearing impairment and the prevalence of mental disorders among families during this specific period, but causation cannot be definitively established in this observational cross-sectional research. Second, despite that the random sampling design was considered to reduce the possibility of selection bias, variations in demographics, random selection, and response rates from year to year may introduce certain inconsistencies that cannot be totally eliminated. In addition, as all the results are self-reported by the respondents, recall bias and reporting errors could inevitably be introduced. Another limitation is the imperfection of information. Due to data limitations, we lack specific information regarding the circumstances of hearing impairment in children; this includes hearing thresholds, interventions, and prognosis in the year of onset, as well as the duration of the hearing impairment. Additionally, though multivariate logistic regression analyses and subgroup analyses were utilized to control the potential confounding factors, some factors, such as the health status of other children or family members, were not controlled due to the resolution and granularity of the data. This could lead to confounding bias and become a source of error in calculating associations/ odds ratios. Consequently, we can only draw relatively vague conclusions and cannot define the extent to which children’s hearing impairment causes anxiety and depression. Further research is needed to provide a more subtle understanding of this relationship.

## Conclusion

In brief, this study reaffirms the substantial mental health burden experienced by hearing-impaired children as well as their parents. To our knowledge, this is the first research using a nationally representative survey to take a holistic view of families of children with hearing-impaired children and explore the impact of hearing impairment on both children’s and their parents’ mental outcomes. It reveals that this burden is influenced by several internal and external variables, some of which have been rarely addressed in previous studies. Families of hearing-impaired children appear to be in a vulnerable position during public health crises such as the COVID-19 pandemic, which can further exacerbate their mental outcomes. We call for improvement in psycho-social support for families with hearing-impaired children in routine and emergency situations.

## Data Availability

Publicly available datasets were analyzed in this study. This data can be found here: https://www.cdc.gov/nchs/nhis/data-questionnaires-documentation.htm.

## Footnotes

^1^https://www.cdc.gov/nchs/nhis/index.htm
