# Supplementary material for: Psychological burden of hearing-impaired children and their parents through the COVID-19 pandemic
Source: Front Public Health. 2024 Sep 17;12:1403729. doi: 10.3389/fpubh.2024.1403729 (PMC11442390; doi:10.3389/fpubh.2024.1403729)
Supplement: Supplementary file 1 [file Data_Sheet_1.DOCX]

**Supplementary Method**

The questions and answer choices of mental outcomes and health care utilization during the COVID-19 pandemic used in this research. (The entire survey and dataset are publicly available at [https://www.cdc.gov/nchs/nhis/data-questionnaires-documentation.htm.)](https://www.cdc.gov/nchs/nhis/data-questionnaires-documentation.htm))

1. Children’s anxiety frequency —— “ANXFREQ_C”

Question: How often does the child seem very anxious, nervous, or worried? Would you say: daily, weekly, monthly, a few times a year, or never?

Answer choices:

- Daily
- Weekly
- Monthly
- A few times a year
- Never
- Refused
- Not Ascertained
- Don’t know

1. Children’s depression frequency —— “DEPFREQ_C”

Question: How often does the child seem very sad or depressed? Would you say: daily, weekly, monthly, a few times a year, or never?

Answer choices:

- Daily
- Weekly
- Monthly
- A few times a year
- Never
- Refused
- Not Ascertained
- Don’t know

1. Parental anxiety disorder —— “ANXEV_A”

Question: Have you EVER been told by a doctor or other health professional that you had any type of anxiety disorder?

*Some common types of anxiety disorders include generalized anxiety disorder, social anxiety disorder, panic disorder, posttraumatic stress disorder, obsessive-compulsive disorder, and phobias.

Answer choices:

- Yes
- No
- Refused
- Not Ascertained
- Don’t Know

1. Parental anxiety frequency: “ANXFREQ_A”

Question: How often do you feel worried, nervous or anxious? Would you say daily, weekly, monthly, a few times a year, or never?

*If respondent asks whether they are to answer about their emotional state after taking mood-regulating medications, say: “Please answer based on your usual use of medication.

Answer choices:

- Daily
- Weekly
- Monthly
- A few times a year
- Never
- Refused
- Not Ascertained
- Don’t know

1. Parental depression disorder: “DEPEV_A”

Question: Have you EVER been told by a doctor or other health professional that you had any type of depression?

*Some common types of depression include major depression (or major depressive disorder), bipolar depression, dysthymia, post-partumdepression, and seasonal affective disorder.

Answer choices:

- Yes
- No
- Refused
- Not Ascertained
- Don’t Know

1. Parental depression frequency: “DEPFREQ_A”

Question: How often do you feel depressed? Would you say daily, weekly, monthly, a few times a year, or never?

*If respondent asks whether they are to answer about their emotional state after taking mood-regulating medications, say: “Please answer based on your usual use of medication.

Answer choices:

- Daily
- Weekly
- Monthly
- A few times a year
- Never
- Refused
- Not Ascertained
- Don’t know

1. Delayed medical care: “DLYCARE_C”

Was there any time when the child DELAYED getting medical care because of the coronavirus pandemic?

Answer choices:

- Yes
- No
- Refused
- Not Ascertained
- Don’t Know

1. Absent medical care: “DNGCARE_C”

Question: Was there any time when the child needed medical care for something other than coronavirus, but DID NOT GET IT because of the coronavirus pandemic?

Answer choices:

- Yes
- No
- Refused
- Not Ascertained
- Don’t Know

**Supplementary Table**

**Supplementary Table 1.** Association between childhood hearing impairment and children’s higher frequency of anxiety in subgroup analysis, 2019-2022 NHIS

|  | **Hearing impairment versus No hearing impairment** | |  |
| --- | --- | --- | --- |
| **Subgroup** | **OR (95% CI)^a^** | ***P* for interaction** |  |
| **Year of interview** |  | 0.728 |  |
| Before COVID-19 | 2.06 (1.29-3.27) |  |  |
| Peak stage of COVID-19 | 2.45 (1.66-3.61) |  |  |
| Late stage of COVID-19 | 2.37 (1.41-3.98) |  |  |
| **Age of parents** |  | 0.720 |  |
| ≤40 | 2.23 (1.56-3.18) |  |  |
| > 40 | 2.47 (1.70-3.57) |  |  |
| **Sex of parents** |  | 0.284 |  |
| Male | 2.80 (1.86-4.20) |  |  |
| Female | 2.12 (1.52-2.97) |  |  |
| **Educational level of parents** |  | 0.985 |  |
| High school or below | 2.75 (1.78-4.26) |  |  |
| Associate degree or some college | 1.63 (1.01-2.64) |  |  |
| Bachelor's degree or above | 2.64 (1.72-4.04) |  |  |
| **Race group of parents** |  | 0.007** |  |
| White only | 2.00 (1.50-2.66) |  |  |
| Black/African American only | 4.11 (1.93-8.75) |  |  |
| Others | 4.47 (2.20-9.09) |  |  |
| **Hearing impairment of parents** |  | 0.756 |  |
| No impairment | 2.38 (1.80-3.15) |  |  |
| Hearing impairment | 2.26 (1.22-4.17) |  |  |
| **PIR** |  | 0.178 |  |
| <2 | 2.61 (1.71-3.99) |  |  |
| 2-4 | 2.89 (1.86-4.48) |  |  |
| >4 | 1.73 (1.11-2.69) |  |  |
| **Number of adults in the family** |  | 0.365 |  |
| 1 | 2.70 (1.30-5.59) |  |  |
| 2 | 2.43 (1.74-3.40) |  |  |
| 3+ | 1.85 (1.05-3.25) |  |  |
| **Number of children in the family** |  | 0.244 |  |
| 1 | 3.16 (2.10-4.76) |  |  |
| 2 | 2.17 (1.43-3.28) |  |  |
| 3+ | 2.19 (1.38-3.49) |  |  |
| **Age of children** |  | 0.265 |  |
| ≤12 | 2.05 (1.44-2.91) |  |  |
| >12 | 2.85 (1.95-4.16) |  |  |
| **Sex of children** |  | 0.815 |  |
| Male | 2.28 (1.58-3.30) |  |  |
| Female | 2.49 (1.76-3.51) |  |  |
| **Race group of children** |  | 0.048* |  |
| White only | 2.04 (1.52-2.73) |  |  |
| Black/African American only | 3.79 (1.71-8.42) |  |  |
| Others | 3.44 (1.70-6.93) |  |  |

Abbreviation: OR, odds ratio; CI, confidence interval; PIR, Ratio of family income to poverty threshold.

^a^Adjusted for age of children, sex of children, race group of children, the number of adults in the family, the number of children in the family, and ratio of family income to poverty threshold (PIR), the age of the parents, sex of the parents, educational level of the parents, race group of the parents, and the presence of hearing impairment in the parents.

NOTES: Socioeconomic demographic factors that exhibit significant interaction effect with childhood hearing loss on children’s and parental anxiety and depression outcomes (all *P* for interaction < 0.1) were selected and depicted in **Figure 1** of the main text.

^•^*P* < .1.

**P*< .05.

** p < .01.

**Supplementary Table 2.** Association between childhood hearing impairment and children’s higher frequency of depression in subgroup analysis, 2019-2022 NHIS

|  | **Hearing impairment versus No hearing impairment** | |  |
| --- | --- | --- | --- |
| **Subgroup** | **OR (95% CI)^a^** | ***P* for interaction** |  |
| **Year of interview** |  | 0.428 |  |
| Before COVID-19 | 1.91 (1.05-3.46) |  |  |
| Peak stage of COVID-19 | 1.91 (1.21-3.03) |  |  |
| Late stage of COVID-19 | 2.80 (1.63-4.83) |  |  |
| **Age of parents** |  | 0.969 |  |
| ≤40 | 2.10 (1.38-3.19) |  |  |
| >40 | 2.18 (1.41-3.37) |  |  |
| **Sex of parents** |  | 0.947 |  |
| Male | 2.15 (1.32-3.48) |  |  |
| Female | 2.12 (1.52-2.97) |  |  |
| **Educational level of parents** |  | 0.764 |  |
| High school or below | 2.30 (1.41-3.74) |  |  |
| Associate degree or some college | 1.91 (1.10-3.29) |  |  |
| Bachelor's degree or above | 2.36 (1.43-3.90) |  |  |
| **Race group of parents** |  | 0.351 |  |
| White only | 1.99 (1.43-2.77) |  |  |
| Black/African American only | 2.85 (1.02-7.92) |  |  |
| Others | 3.48 (1.38-8.77) |  |  |
| **Hearing impairment of parents** |  | 0.827 |  |
| No impairment | 2.18 (1.55-3.05) |  |  |
| Hearing impairment | 2.00 (1.04-3.88) |  |  |
| **PIR** |  | 0.609 |  |
| <2 | 2.29 (1.47-3.57) |  |  |
| 2-4 | 2.46 (1.42-4.24) |  |  |
| >4 | 1.88 (1.08-3.28) |  |  |
| **Number of adults in the family** |  | 0.480 |  |
| 1 | 2.49 (1.24-5.02) |  |  |
| 2 | 2.11 (1.41-3.17) |  |  |
| 3+ | 1.93 (1.03-3.62) |  |  |
| **Number of children in the family** |  | 0.634 |  |
| 1 | 2.70 (1.65-4.44) |  |  |
| 2 | 1.91 (1.19-3.09) |  |  |
| 3+ | 2.06 (1.19-3.58) |  |  |
| **Age of children** |  | 0.515 |  |
| ≤12 | 1.86 (1.22-2.86) |  |  |
| >12 | 2.37 (1.55-3.61) |  |  |
| **Sex of children** |  | 0.804 |  |
| Male | 2.02 (1.33-3.08) |  |  |
| Female | 2.26 (1.49-3.41) |  |  |
| **Race group of children** |  | 0.286 |  |
| White only | 1.96 (1.39-2.75) |  |  |
| Black/African American only | 3.44 (1.27-9.33) |  |  |
| Others | 2.90 (1.34-6.30) |  |  |

Abbreviation: OR, odds ratio; CI, confidence interval; PIR, Ratio of family income to poverty threshold.

**^a^** Adjusted for age of children, sex of children, race group of children, the number of adults in the family, the number of children in the family, and ratio of family income to poverty threshold (PIR), the age of the parents, sex of the parents, educational level of the parents, race group of the parents, and the presence of hearing impairment in the parents.

NOTES: Socioeconomic demographic factors that exhibit significant interaction effect with childhood hearing loss on children’s and parental anxiety and depression outcomes (all *P* for interaction < 0.1) were selected and depicted in **Figure 1** of the main text.

^•^*P* < .1.

**P*< .05.

** p < .01.

**Supplementary Table 3.** Association between children’s hearing impairment and parental anxiety disorder in subgroup analysis, 2019-2022 NHIS

|  | **Hearing impairment versus No hearing impairment** | |  |
| --- | --- | --- | --- |
| **Subgroup** | **OR (95% CI)^a^** | ***P* for interaction** |  |
| **Year of interview** |  | 0.368 |  |
| Before COVID-19 | 1.73 (1.06-2.83) |  |  |
| Peak stage of COVID-19 | 1.72 (1.13-2.63) |  |  |
| Late stage of COVID-19 | 2.39 (1.42-4.01) |  |  |
| **Age of parents** |  | 0.780 |  |
| ≤40 | 1.97 (1.36-2.86) |  |  |
| >40 | 1.80 (1.20-2.71) |  |  |
| **Sex of parents** |  | 0.705 |  |
| Male | 1.76 (1.04-2.97) |  |  |
| Female | 1.95 (1.41-2.70) |  |  |
| **Educational level of parents** |  | 0.474 |  |
| High school or below | 1.91 (1.17-3.12) |  |  |
| Associate degree or some college | 1.49 (0.93-2.39) |  |  |
| Bachelor's degree or above | 2.38 (1.55-3.64) |  |  |
| **Race group of parents** |  | 0.399 |  |
| White only | 1.81 (1.34-2.46) |  |  |
| Black/African American only | 2.31 (0.96-5.54) |  |  |
| Others | 2.52 (1.10-5.78) |  |  |
| **Hearing impairment of parents** |  | 0.277 |  |
| No impairment | 2.07 (1.55-2.78) |  |  |
| Hearing impairment | 1.32 (0.70-2.51) |  |  |
| **PIR** |  | 0.140 |  |
| <2 | 2.14 (1.40-3.29) |  |  |
| 2-4 | 2.51 (1.60-3.95) |  |  |
| >4 | 1.18 (0.67-2.07) |  |  |
| **Number of adults in the family** |  | 0.408 |  |
| 1 | 2.58 (1.34-4.96) |  |  |
| 2 | 1.74 (1.22-2.50) |  |  |
| 3+ | 1.70 (0.85-3.40) |  |  |
| **Number of children in the family** |  | 0.806 |  |
| 1 | 1.96 (1.26-3.06) |  |  |
| 2 | 1.90 (1.23-2.95) |  |  |
| 3+ | 1.86 (1.13-3.07) |  |  |
| **Age of children** |  | 0.662 |  |
| ≤12 | 2.00 (1.40-2.86) |  |  |
| >12 | 1.77 (1.14-2.74) |  |  |
| **Sex of children** |  | 0.629 |  |
| Male | 1.78 (1.20-2.63) |  |  |
| Female | 2.02 (1.36-3.01) |  |  |
| **Race group of children** |  | 0.058^•^ |  |
| White only | 1.73 (1.26-2.38) |  |  |
| Black/African American only | 1.62 (0.62-4.24) |  |  |
| Others | 3.95 (2.16-7.22) |  |  |

Abbreviation: OR, odds ratio; CI, confidence interval; PIR, Ratio of family income to poverty threshold.

**^a^** Adjusted for age of children, sex of children, race group of children, the number of adults in the family, the number of children in the family, and ratio of family income to poverty threshold (PIR), the age of the parents, sex of the parents, educational level of the parents, race group of the parents, and the presence of hearing impairment in the parents.

NOTES: Socioeconomic demographic factors that exhibit significant interaction effect with childhood hearing loss on children’s and parental anxiety and depression outcomes (all *P* for interaction < 0.1) were selected and depicted in **Figure 1** of the main text.

^•^*P* < .1.

**P*< .05.

** p < .01.

**Supplementary Table 4.** Association between children’s hearing impairment and parental higher frequency of anxiety in subgroup analysis, 2019-2022 NHIS

|  | **Hearing impairment versus No hearing impairment** | |
| --- | --- | --- |
| **Subgroup** | **OR (95% CI)^a^** | ***P* for interaction** |
| **Year of interview** |  | 0.251 |
| Before COVID-19 | 1.15 (0.73-1.79) |  |
| Peak stage of COVID-19 | 1.73 (1.16-2.59) |  |
| Late stage of COVID-19 | 1.67 (0.98-2.85) |  |
| **Age of parents** |  | 0.565 |
| ≤40 | 1.65 (1.16-2.37) |  |
| >40 | 1.45 (1.00-2.09) |  |
| **Sex of parents** |  | 0.068^•^ |
| Male | 1.11 (0.72-1.69) |  |
| Female | 1.87 (1.34-2.63) |  |
| **Educational level of parents** |  | 0.948 |
| High school or below | 1.53 (0.98-2.40) |  |
| Associate degree or some college | 1.78 (1.14-2.76) |  |
| Bachelor's degree or above | 1.46 (0.96-2.24) |  |
| **Race group of parents** |  | 0.123 |
| White only | 1.41 (1.05-1.88) |  |
| Black/African American only | 1.91 (0.87-4.20) |  |
| Others | 2.33 (1.13-4.83) |  |
| **Hearing impairment of parents** |  | 0.197 |
| No impairment | 1.69 (1.28-2.24) |  |
| Hearing impairment | 1.02 (0.52-1.98) |  |
| **PIR** |  | 0.438 |
| <2 | 1.59 (1.03-2.46) |  |
| 2-4 | 2.10 (1.35-3.26) |  |
| >4 | 1.18 (0.77-1.82) |  |
| **Number of adults in the family** |  | 0.198 |
| 1 | 2.60 (1.13-5.98) |  |
| 2 | 1.45 (1.05-1.99) |  |
| 3+ | 1.28 (0.73-2.25) |  |
| **Number of children in the family** |  | 0.347 |
| 1 | 1.56 (1.02-2.41) |  |
| 2 | 1.93 (1.31-2.85) |  |
| 3+ | 1.33 (0.84-2.12) |  |
| **Age of children** |  | 0.184 |
| ≤12 | 1.80 (1.26-2.58) |  |
| >12 | 1.24 (0.84-1.82) |  |
| **Sex of children** |  | 0.562 |
| Male | 1.43 (0.98-2.09) |  |
| Female | 1.70 (1.17-2.46) |  |
| **Race group of children** |  | 0.012* |
| White only | 1.33 (0.99-1.80) |  |
| Black/African American only | 1.74 (0.77-3.94) |  |
| Others | 3.16 (1.74-5.72) |  |

Abbreviation: OR, odds ratio; CI, confidence interval; PIR, Ratio of family income to poverty threshold.

**^a^** Adjusted for age of children, sex of children, race group of children, the number of adults in the family, the number of children in the family, and ratio of family income to poverty threshold (PIR), the age of the parents, sex of the parents, educational level of the parents, race group of the parents, and the presence of hearing impairment in the parents.

NOTES: Socioeconomic demographic factors that exhibit significant interaction effect with childhood hearing loss on children’s and parental anxiety and depression outcomes (all *P* for interaction < 0.1) were selected and depicted in **Figure 1** of the main text.

^•^*P* < .1.

**P*< .05.

** p < .01.

**Supplementary Table 5.** Association between children’s hearing impairment and parental depression disorder in subgroup analysis, 2019-2022 NHIS

|  | **Hearing impairment versus No hearing impairment** | |
| --- | --- | --- |
| **Subgroup** | **OR (95% CI)^a^** | ***P* for interaction** |
| **Year of interview** |  | 0.036* |
| Before COVID-19 | 1.00 (0.58-1.74) |  |
| Peak stage of COVID-19 | 1.91 (1.24-2.95) |  |
| Late stage of COVID-19 | 2.24 (1.31-3.84) |  |
| **Age of parents** |  | 0.482 |
| ≤40 | 1.57 (1.06-2.33) |  |
| >40 | 1.99 (1.31-3.00) |  |
| **Sex of parents** |  | 0.808 |
| Male | 1.81 (1.08-3.03) |  |
| Female | 1.69 (1.21-2.37) |  |
| **Educational level of parents** |  | 0.900 |
| High school or below | 1.85 (1.14-3.00) |  |
| Associate degree or some college | 1.72 (1.08-2.74) |  |
| Bachelor's degree or above | 1.67 (1.05-2.65) |  |
| **Race group of parents** |  | 0.863 |
| White only | 1.76 (1.27-2.44) |  |
| Black/African American only | 1.57 (0.62-4.02) |  |
| Others | 1.41 (0.61-3.27) |  |
| **Hearing impairment of parents** |  | 0.971 |
| No impairment | 1.71 (1.25-2.35) |  |
| Hearing impairment | 1.64 (0.83-3.24) |  |
| **PIR** |  | 0.431 |
| <2 | 2.07 (1.33-3.24) |  |
| 2-4 | 1.24 (0.76-2.02) |  |
| >4 | 1.62 (0.93-2.83) |  |
| **Number of adults in the family** |  | 0.190 |
| 1 | 1.68 (0.88-3.20) |  |
| 2 | 1.44 (0.97-2.12) |  |
| 3+ | 2.87 (1.61-5.11) |  |
| **Number of children in the family** |  | 0.372 |
| 1 | 2.00 (1.29-3.09) |  |
| 2 | 1.86 (1.16-2.98) |  |
| 3+ | 1.42 (0.84-2.41) |  |
| **Age of children** |  | 0.583 |
| ≤12 | 1.80 (1.23-2.63) |  |
| >12 | 1.56 (1.00-2.45) |  |
| **Sex of children** |  | 0.888 |
| Male | 1.70 (1.14-2.54) |  |
| Female | 1.76 (1.16-2.67) |  |
| **Race group of children** |  | 0.438 |
| White only | 1.85 (1.33-2.58) |  |
| Black/African American only | 1.46 (0.54-3.93) |  |
| Others | 1.16 (0.51-2.65) |  |

Abbreviation: OR, odds ratio; CI, confidence interval; PIR, Ratio of family income to poverty threshold.

**^a^** Adjusted for age of children, sex of children, race group of children, the number of adults in the family, the number of children in the family, and ratio of family income to poverty threshold (PIR), the age of the parents, sex of the parents, educational level of the parents, race group of the parents, and the presence of hearing impairment in the parents.

NOTES: Socioeconomic demographic factors that exhibit significant interaction effect with childhood hearing loss on children’s and parental anxiety and depression outcomes (all *P* for interaction < 0.1) were selected and depicted in **Figure 1** of the main text.

^•^*P* < .1.

**P*< .05.

** p < .01.

**Supplementary Table 6.** Association between children’s hearing impairment and parental higher frequency of depression in subgroup analysis, 2019-2022 NHIS

|  | **Hearing impairment versus No hearing impairment** | |
| --- | --- | --- |
| **Subgroup** | **OR (95% CI)^a^** | ***P* for interaction** |
| **Year of interview** |  | 0.067^•^ |
| Before COVID-19 | 1.31 (0.81-2.10) |  |
| Peak stage of COVID-19 | 1.64 (1.05-2.57) |  |
| Late stage of COVID-19 | 2.43 (1.46-4.05) |  |
| **Age of parents** |  | 0.856 |
| ≤40 | 1.67 (1.14-2.45) |  |
| >40 | 1.84 (1.19-2.85) |  |
| **Sex of parents** |  | 0.613 |
| Male | 1.57 (0.97-2.54) |  |
| Female | 1.81 (1.28-2.55) |  |
| **Educational level of parents** |  | 0.143 |
| High school or below | 1.96 (1.21-3.19) |  |
| Associate degree or some college | 2.24 (1.41-3.55) |  |
| Bachelor's degree or above | 1.06 (0.64-1.76) |  |
| **Race group of parents** |  | 0.914 |
| White only | 1.77 (1.28-2.44) |  |
| Black/African American only | 1.25 (0.50-3.10) |  |
| Others | 2.04 (0.95-4.36) |  |
| **Hearing impairment of parents** |  | 0.448 |
| No impairment | 1.61 (1.18-2.21) |  |
| Hearing impairment | 2.13 (1.10-4.10) |  |
| **PIR** |  | 0.033 |
| <2 | 2.20 (1.43-3.40) |  |
| 2-4 | 2.04 (1.30-3.19) |  |
| >4 | 0.80 (0.37-1.72) |  |
| **Number of adults in the family** |  | 0.466 |
| 1 | 1.77 (0.94-3.34) |  |
| 2 | 1.54 (1.05-2.27) |  |
| 3+ | 2.19 (1.23-3.89) |  |
| **Number of children in the family** |  | 0.811 |
| 1 | 1.65 (1.07-2.54) |  |
| 2 | 1.94 (1.27-2.95) |  |
| 3+ | 1.58 (0.93-2.67) |  |
| **Age of children** |  | 0.478 |
| ≤12 | 1.87 (1.28-2.74) |  |
| >12 | 1.51 (1.01-2.26) |  |
| **Sex of children** |  | 0.132 |
| Male | 1.43 (0.95-2.14) |  |
| Female | 2.17 (1.46-3.22) |  |
| **Race group of children** |  | 0.607 |
| White only | 1.86 (1.34-2.57) |  |
| Black/African American only | 1.16 (0.44-3.06) |  |
| Others | 1.55 (0.76-3.15) |  |

Abbreviation: OR, odds ratio; CI, confidence interval; PIR, Ratio of family income to poverty threshold.

**^a^** Adjusted for age of children, sex of children, race group of children, the number of adults in the family, the number of children in the family, and ratio of family income to poverty threshold (PIR), the age of the parents, sex of the parents, educational level of the parents, race group of the parents, and the presence of hearing impairment in the parents.

NOTES: Socioeconomic demographic factors that exhibit significant interaction effect with childhood hearing loss on children’s and parental anxiety and depression outcomes (all *P* for interaction < 0.1) were selected and depicted in **Figure 1** of the main text.

^•^*P* < .1.

**P*< .05.

** p < .01.

**Supplementary Table 7.** Characteristics of the study population, 2020 (quarter 3 and quarter 4)-2021 NHIS

| **Variables** | **Without hearing**  **impairment** | **With hearing impairment** | ***P* value** |
| --- | --- | --- | --- |
| Total (%, weighted n) | 97.9 (27619808.7)^a^ | 2.1 (589553.1)^a^ |  |
| **Parental characteristics** |  |  |  |
| Age (%) |  |  | 0.089 |
| ≤40 | 49.1 | 58.0 |  |
| >40 | 50.9 | 42.0 |  |
| Sex (%) |  |  | 0.217 |
| Male | 41.6 | 34.9 |  |
| Female | 58.4 | 65.1 |  |
| Educational level (%) |  |  | 0.194 |
| High school or below | 28.9 | 37.9 |  |
| Associate degree or some college | 29.2 | 27.3 |  |
| Bachelor's degree or above | 41.8 | 34.8 |  |
| Race group (%) |  |  | 0.480 |
| White only | 75.2 | 74.7 |  |
| Black/African American only | 14.6 | 18.1 |  |
| Others^b^ | 10.2 | 7.1 |  |
| Hearing impairment (%) |  |  | <0.001*** |
| No impairment | 91.3 | 79.8 |  |
| Hearing impairment | 8.7 | 20.2 |  |
| **Family’s characteristics** |  |  |  |
| PIR (%) |  |  | 0.182 |
| >2 | 34.8 | 43.2 |  |
| 2-4 | 28.7 | 29.3 |  |
| >4 | 36.5 | 27.5 |  |
| Number of adults in the family (%) |  |  | 0.281 |
| 1 | 17.2 | 23.1 |  |
| 2 | 65.3 | 63.2 |  |
| 3+ | 17.5 | 13.7 |  |
| Number of children in the family (%) |  |  | 0.047* |
| 1 | 20.5 | 31.4 |  |
| 2 | 41.8 | 31.8 |  |
| 3+ | 37.7 | 36.7 |  |
| **Children’s characteristics** |  |  |  |
| Age (%) |  |  | 0.635 |
| ≤12 | 61.6 | 64.2 |  |
| >12 | 38.4 | 35.8 |  |
| Sex (%) |  |  | 0.566 |
| Male | 50.4 | 47.1 |  |
| Female | 49.6 | 52.9 |  |
| Race group (%) |  |  | 0.631 |
| White only | 72.4 | 67.8 |  |
| Black/African American only | 14.2 | 18.0 |  |
| Others^b^ | 13.5 | 14.2 |  |

Abbreviation: PIR, Ratio of family income to poverty threshold.

^a^ A total of 3,641 families were eligible for inclusion, of which 423 (11.16%) were excluded due to incomplete data. Unweighted number of families with hearing impaired children was 116 and the number of families without hearing impaired children was 5,191.

^b^ Other race groups include responses of “Asian only”, “American Indian and Alaska Native [AIAN] only”, “AIAN and any other group”, and “Other single and multiple races”.

NOTES: Variables concerning the COVID-19 pandemic started to field in the NHIS since quarter 3 of 2020.

*p < .05.

** p < .01.

*** p < .001.
